# Phytochemical Analysis and Potential Biological Activities of Essential Oil from Rice Leaf

**DOI:** 10.3390/molecules24030546

**Published:** 2019-02-02

**Authors:** Truong Ngoc Minh, Tran Dang Xuan, Truong Mai Van, Yusuf Andriana, Tran Duc Viet, Tran Dang Khanh, Hoang-Dung Tran

**Affiliations:** 1 Graduate school for International Development and Cooperation, Hiroshima University, Hiroshima 739-8529, Japan; minhtn689@gmail.com (T.N.M.); truongmaivan1991@gmail.com (T.M.V.); yusufandriana@yahoo.com (Y.A.); viettran1609@gmail.com (T.D.V.); 2 Agricultural Genetics Institute, Hanoi City 123000, Vietnam; tdkhanh@vaas.vn; 3 Center for Expert, Vietnam National University of Agriculture, Hanoi 131000, Vietnam; 4 Department of Biotechnology, NTT Institute of Hi-Technology, Nguyen-Tat-Thanh University, 298A-300A Nguyen-Tat-Thanh Street, 13 Ward, District 04, Ho Chi Minh City 72820, Vietnam

**Keywords:** Momilactone A, momilactone B, antioxidant activity, allelopathic activity, anti-hyperuricemia, rice leaf, essential, UPLC/ESI-MS

## Abstract

Although many investigations on phytochemicals in rice plant parts and root exudates have been conducted, information on the chemical profile of essential oil (EO) and potent biological activities has been limited. In this study, chemical compositions of rice leaf EO and in vitro biological activities were investigated. From 1.5 kg of fresh rice leaves, an amount of 20 mg EO was obtained by distillation and analyzed by gas chromatography-mass spectrometry (GC-MS), electrospray ionization (ESI), and atmospheric pressure chemical ionization (APCI) to reveal the presence of twelve volatile constituents, of which methyl ricinoleate (27.86%) was the principal compound, followed by palmitic acid (17.34%), and linolenic acid (11.16%), while 2-pentadecanone was the least (2.13%). Two phytoalexin momilactones A and B were first time identified in EO using ultra-performance liquid chromatography coupled with electrospray mass spectrometry (UPLC/ESI-MS) (9.80 and 4.93 ng/g fresh weight, respectively), which accounted for 7.35% and 3.70% of the EO, respectively. The assays of DPPH (IC_50_ = 73.1 µg/mL), ABTS (IC_50_ = 198.3 µg/mL), FRAP (IC_50_ = 700.8 µg/mL) and β-carotene oxidation (LPI = 79%) revealed that EO possessed an excellent antioxidant activity. The xanthine oxidase assay indicated that the anti-hyperuricemia potential was in a moderate level (IC_50_ = 526 µg/mL) as compared with the standard allopurinol. The EO exerted potent inhibition on growth of *Raphanus sativus, Lactuca sativa*, and two noxious weeds *Echinochloa crus-galli*, and *Bidens pilosa*, but in contrast, the growth of rice seedlings was promoted. Among the examined plants, the growth of the *E. crus-galli* root was the most inhibited, proposing that constituents found in EO may have potential for the control of the problematic paddy weed *E. crus-galli*. It was found that the EO of rice leaves contained rich phytochemicals, which were potent in antioxidants and gout treatment, as well as weed management. Findings of this study highlighted the potential value of rice leaves, which may provide extra benefits for rice farmers.

## 1. Introduction

The plant kingdom abounds with natural products which are chemically diverse [1]. During growth and development, plants generate around 100,000 chemical products, of which, 1700 are volatile organic compounds (VOC) [2] presented in essential oils. These molecules are emitted by different plant organs including leaves, flowers, fruits, and roots [2,3]. Plant VOCs are chemically classified into different groups such as fatty acid derivatives, terpenes, indole, and molecules from other chemical families. Biologically, volatile compounds are not only to protect themselves from herbivores and microbial pathogens, but they also release signals and messages to insects and neighbouring plants [3]. Due to having therapeutic benefits and high absorbability through the skin, VOCs and essential oils have been widely applied in different sectors including food, cosmetic, and medicinal production [4]. From an agricultural point of view, such constituents play a notable role in enhancing crop protection [5].

Rice (*Oryza sativa* L.), which belongs to the *Gramineae* family, has been consumed by humans for almost 5000 years. Rice is a major crop used by two-thirds of the population over the world as a staple food [6]. Instrumental analyses have found over 200 volatile compounds presented in rice [7] and usually distributed in rice leaves and rice bran [8]. Among them, rice diterpenoids such as momilactones and oryzalexins play vital functions as phytohormones and phytoalexins. Most of the previous studies have investigated on VOCs of rice grains of different varieties [9], the changes of VOCs during storage and cooked process [10,11,12,13,14,15,16,17,18,19]. However, little information has been found concerning VOC releasing from rice leaves.

In rice production, abundant rice leaves are available but no potential value, which may provide further income for rice farmers once this has been achieved. Thus, this study aims to investigate the profile of essential oil (EO) in rice leaves and evaluate their potential biological properties including antioxidant, antibacterial, and anti-hyperuricemia activities in vitro assays. The obtained compounds were identified by gas chromatography-mass spectrometry (GC-MS), electrospray ionization (ESI), atmospheric pressure chemical ionization (APCI), and ultra-performance liquid chromatography coupled with electrospray mass spectrometry (UPLC/ESI-MS).

## 2. Results

### 2.1. Antioxidant and Xanthine Oxidase Inhibitory (XOI) Activities of Rice Leaf EO

The antioxidant potential of rice leaf EO was evaluated using the 2, 2-diphenyl-1-picrylhydrazyl (DPPH) and 2,2′-azino-bis(3-ethylbenzothiazoline-6-sulphonic acid) (ABTS) radical scavenging, ferric reducing antioxidant power (FRAP), and β-carotene linoleate assays. The inhibitory effect of EO was compared with a positive standard butylated hydroxytoluene (BHT) (Table 1). The result of β-carotene oxidation assay was expressed as percent inhibition of β-carotene bleaching after 180 min of reaction (79.0%). Whilst that of other tests were presented as IC_50_ values at which concentration requires to inhibit 50% of scavenging free radicals (DPPH (73.1 µg/mL) and ABTS (193.3 µg/mL)) and reducing of ferric iron Fe III in the complex to ferrous iron Fe II (700.8 µg/mL). A small IC_50_ value indicates a superior activity. The obtained data showed that the EO from rice leaf exhibited a strong antioxidant property in all assays, although the synthetic BHT exerted stronger antioxidant level.

The inhibitory effect of rice leaf EO on xanthine oxidase was presented in Table 1. The result showed that the EO from rice leaf exhibited a considerable activity against xanthine oxidase enzyme (IC_50_ = 526.0 µg/mL). Although the anti-hyperuricemia property of allopurinol was superior to that of the tested EO, further examination on the xanthine oxidase inhibition of individual volatile constituents in EO should be elaborated to search for more potent volatiles in rice EO.

### 2.2. Growth Inhibitory Activities of Rice Leaf Essential Oil 

The levels of growth inhibitory effects were varied among *Oryza sativa* (Koshihikari), *Echinochloa crus-galli, Bidens pilosa*, *Raphanus sativus*, and *Lactuca sativa*. Table 2 showed that the EO reduced the shoot and root elongation of the two indicator plants *R. sativus* and *L. sativa*, and the two noxious weeds *E. crus-galli* and *B. pilosa*. The inhibition was proportional to the applied doses of EO (Table 2). In contrast, all applied doses of EO stimulated slightly (5–13%) the growth of the rice.

The IC_50_ parameter was used to compare the inhibitory levels among the examined species, of which the smaller IC_50_ value presented the higher suppressing level. Among the tested plants, the maximum inhibition was observed on the root growth of weed *E. crus-galli* (IC_50_ = 455.6 µg/mL) (Table 2). Statistically, the suppressing capacity on root growth of *B. pilosa*, *R. sativus*, and *L. sativa* was similar as their IC_50_ values were 912.5, 916.3, and 926.7 µg/mL, respectively. In general, the strongest inhibition was found on the shoot of R. sativus, followed by *B. pilosa*, *L. sativa*, and *E. crus-galli*. Their IC_50_ values were 866.2, 869.2, 908.0, and 964.3 µg/mL, respectively.

### 2.3. Identification of Phytochemicals in Rice Leaves EO

The GC-MS analysis had quantitatively identified twelve VOCs as they accounted for 88.28% in the rice leaf EO (Table 3). The structural formulas of those compounds were further confirmed by ESI-MS and APCI-MS (Supplementary Materials Figures S1–S4). Based on the percent peak area of each component, methyl ricinoleate (27.86%) was the most dominant compound among volatile oil in rice leaves, followed by palmitic acid (17.34%), and linolenic acid (11.16%), while 2-pentadecanone was the least (2.13%).

### 2.4. Identification and Quantification of MA and MB from Rice Leaf EO by UPLC/ESI–MS Analysis

**Figure 1:**
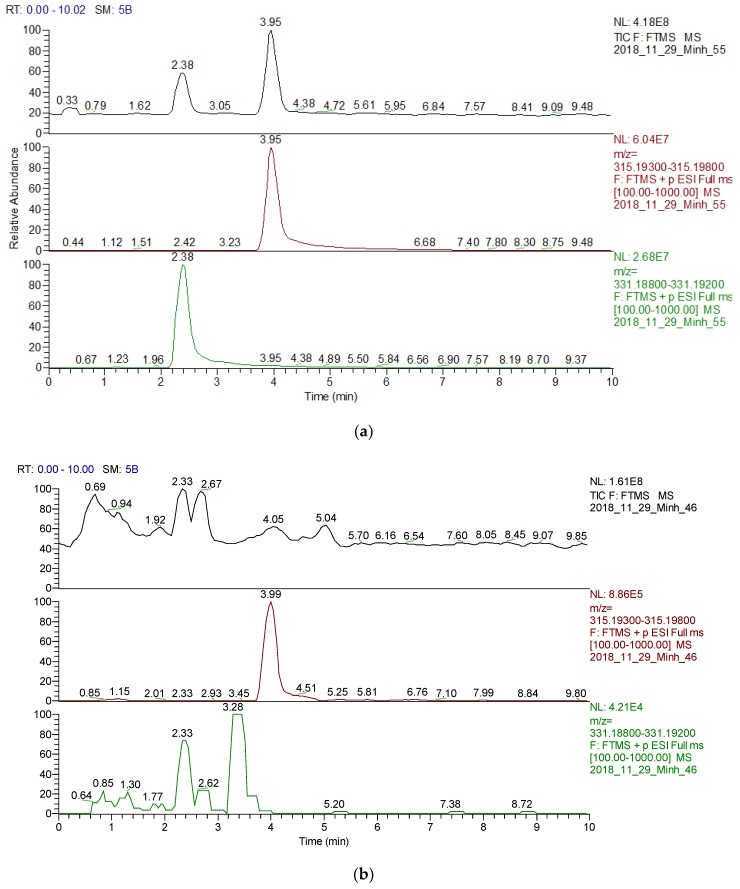
(**a**) UPLC/ESI–MS chromatogram of MA and MB (standard); (**b**) UPLC/ESI–MS chromatogram of MA and MB detected in rice leaf essential oil.

The presence of two momilactones MA and MB were identified and confirmed by UPLC/ESI–MS analysis (Supplementary Materials Figure S5a,b), compared with the standard MA and MB isolated in our laboratories as described previously [20,21]. From 20 mg of rice leaf EO, the amounts of MA and MB detected by this method were 9.80 and 4.93 ng/g fresh weight (FW), respectively. Furthermore, the limit of detection and quantification (LOD and LOQ) parameters were also determined as 0.097, 0.293 ng/mL for MA and 0.157, 0.476 ng/mL for MB, respectively (Table 4, Figure 1a,b).

## 3. Discussion

In this study, the obtained yield of rice leaf EO was 13.33 µg/g fresh weight (FW). This yield is higher than essential oil extracted from *Tagetes erecta* L. leaves (0.72 µg/g FW) [22] but lower than common EO such as *Ocimum basilicum* L. (170–2100 µg/g FW) [23]. EtOAc is the common solvent to extract essential oils from plants [24]; therefore it was used to extract the EO in our research. However, the use of room temperature (25 °C) during the evaporation might result in the yield loss of EO. The yield of EO depends on EO contents in plant tissues and method of extractions [25]. Steam distillation is the most prevalent method of EO extraction [26,27]. The use of lower temperature during evaporation should be examined to enhance the yield of EO in rice leaves. Because rice EO showed potent biological activities as observed by this study, the breeding of new rice cultivars rich with EO may be beneficial to exploit the potent use of rice leaves.

Biological activities of the EO obtained from rice leaves and chemical components were investigated in this study by in vitro assays and spectroscopic analyses. Our results showed that the EO inhibited xanthine oxidase activity by 50% at 526 µg/mL. Additionally, the antioxidant property of the EO was compared with the positive standard BHT in assays DPPH, ABTS, FRAP and β-carotene oxidation (Table 1). It was found that the rice leaf EO was a potent anti-hyperuricemia source and an effective scavenger of superoxidase radicals, although its potency was lower than that of the reference standards (allopurinol and BHT, respectively). Moreover, having both anti-hyperuricemia and antioxidant properties, the rice leaf EO may be well developed as a promising treatment for gout arthritis [28].

The inhibitory activity on the growth of *O. sativa*, *E. crus-galli*, *B. pilosa*, *R. sativus*, and *L. sativa* was also investigated. The reduction observed in the shoot and root lengths of *E. crus-galli*, *B. pilosa*, *R. sativus*, and *L. sativa* (as compared with controls) suggested that the EO exerted toxicity effects on the growth of those tested plants. Among them, the inhibition of the EO on root elongation of *E. crus-galli* was about two-fold stronger than that of *B. pilosa*, *R. sativus*, and *L. sativa*. Interestingly, the EO obtained from rice leaves showed a slight promotion on its own growth. By treating with the EO 1000 µg/mL, the root and shoot of rice plants were increased 13% and 11%, respectively. The results indicated that the rice leaf EO might be highly applicable not only to manage the problematic weed *E. crus-galli* but also to enhance the growth of rice plants in crop production.

EOs are aromatic oily liquids obtained from plant materials. The chemical constituents of EOs are VOCs which can be classified into two major biosynthetic groups including terpenes/terpenoids and aromatic/aliphatic molecules [29,30]. In this work, twelve VOCs of the EO from rice leaves were identified by GC-MS, ESI-MS, and APCI-MS analyses. Although methyl ricinoleate was found in relatively high concentrations (27.86%) of the EO, fatty acids were the most abundant with five components accounting for 36.49%. Among identified volatile acids, palmitic acid was detected in the highest amount (17.34%). These compounds were also encountered in the chemical profile of EOs originating from the rice straw [31], black, and red rice bran [13]. In the literature, methyl ricinoleate was reported as an antioxidant compound, which supports the ethno-medicinal application of *Ricinus communis* seeds extracts in medicine. Additionally, this active metabolite has been considered as an important ingredient in the cosmetic industry, which is used as a plasticizer, lubricant, as emollients, and in skin conditioning or as a fragrance [32]. The compound palmitic acid was previously reported to attribute to antioxidative and antibacterial properties of *Labisia pumia* Benth leaf [33]. As a consequence, these substances might be contributing to the observed antioxidant activity of the rice leaf EO.

Momilactones A and B have been known as phytoalexins and allelochemicals found in rice and the moss *Hypnum plumaeforme* [34,35]. Due to the fact that isolation and purification of the two phytoalexins are laborious and complicated, only few laboratories worldwide have worked with momilactones A and B thus far [20,21,34]. Therefore, those compounds might not be available in the library of JEOL’s GC-MS Mass Center System Version 2.65a and not detectable by common analytical instruments such as GC-MS, ESI-MS, and APIC-MS methods. Because the boiling points of momilactones A and B were 460.1 °C and 504.5 °C, respectively, which exceeded the limited operating temperature of GC (300 °C) that could be detected by the GC-MS. As a consequence, ionizing electrons were not achieved, and fragments could not be produced by molecular ion. By contrast, the replacement of those common spectrometric techniques by UPLC/ESI-MS analysis led to successfully confirming the presence of momilactones A and B in EO of rice leaves. It can be explained by the separation and quantification in UPLC coupled with ESI was performed under very high pressure (up to 15,000 psi, or double as compared with HPLC) in the mobile phase and column design of small material particle size (1.7 µm) in the stationary phase. This analytical technique might be helpful to acquire better resolution, speed, and sensitivity in the analytical process. The presence of momilactones A and B may be responsible for the inhibitory effects of the rice leaf EO on the growth of examined species in this study including *L. sativa*, *R. sativus*, and the two noxious weeds *E. crus-galli* and *B. pilosa*, although they presented in low quantities (9.80 and 4.93 ng/g fresh weight, respectively) (Table 1). This finding was in agreement with previous reports, which documented that amounts of MA in rice plant parts and root exudates was generally greater than MB [20,21,34].

## 4. Materials and Methods 

### 4.1. Plant Materials

Fresh rice leaves were collected from rice (*Oryza sativa* L. cv. Koshihikari) paddy fields close to Hiroshima University, Higashi Hiroshima City, Hiroshima Prefecture, Japan, in August 2017. The samples were kept in sealed nylon bags, cleaned and sterilized at the Laboratory of Plant Physiology and Biochemistry, Hiroshima University, for further analyses.

### 4.2. Preparation of Essential Oil 

**Figure 2:**
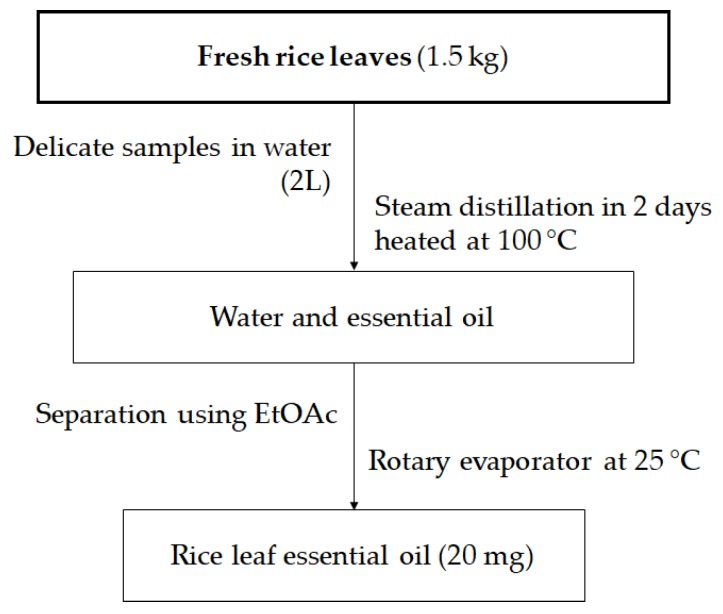
Process of rice leaf essential oil extraction.

Extraction of EO from rice leaves was conducted by the steam distillation method described by Charles and Simon [36] with minor modifications (Figure 2). The apparatus of extracting EO by steam distillation is made up of a heat source, a pear-shaped glass (PSG), and a spherical glass container (SGC) with upper and bottom entrances, a straight glass condenser, and a glass collector for separating and recovering the essential oil; oil appears on top of the water in the collector. A total amount of 1.5 kg fresh rice leaves mixed with 5% (*w*/*v*) sodium chloride solution was sonicated using an ultrasonic cleaner (Branson 5510R-MT Ultrasonic Cleaner, Branson Ultrasonics Corporation, CT 06813, Danbury, CT, USA) for 30 min. The sterilized sample and 2L of water were then placed together in a pear-shaped glass container. The mixture was then heated at 100 °C for 2 days. Consequently, vapor drags the essential oil and is condensed and recovered in the glass collector. The mixture was then extracted using ethyl acetate (EtOAc) to separate EO from the water by a reparatory funnel. Finally, EtOAc extract was evaporated using a rotary evaporator at room temperature (25 °C) to produce 20 mg of rice leaf EO. In each test, an amount of 10 mg EO from fresh rice leaves was used (0.75 kg).

### 4.3. Antioxidant Assays

#### 4.3.1. DPPH Radical Scavenging Assay

The antioxidant activity of essential oil was measured by the method described by Minh et al. [37]. The reaction consisted of 0.08 mL essential oil (10–100 µg/mL in MeOH) and 0.04 mL 0.5 mM DPPH and 0.08 mL of 0.1 M acetate buffer (pH 5.5). The mixtures were incubated in the dark at room temperature for 30 min. The reaction absorbances were read at 517 nm by using a microplate reader (MultiskanTM Microplate Spectrophotometer, Thermo Fisher Scientific, Osaka, Japan). BHT standard (0.01-0.05 mg/mL) was used as a positive control. The DPPH radical scavenging activity of essential oils was calculated as the inhibition percentage by the following equation:

DPPH (%) = [(A_control_ − A_sample_)/A_control_] × 100

A_control_ is the absorbance of reaction without essential oil and A_sample_ is the absorbance of reaction with the sample. The IC_50_ (inhibitory concentration) value is expressed as the concentration of essential oil required to scavenge 50% of DPPH. Lower IC_50_ values indicate higher DPPH radical scavenging activity.

#### 4.3.2. Reducing Power

The reducing power was carried out by using a previous method [38]. 0.1 mL of each essential oil (200-2000 µg/mL in MeOH) or BHT (100-1000 µg/mL) was mixed with 0.25 mL phosphate buffer (0.2M, pH 6.6) and 0.25 mL potassium ferricyanide [K_3_Fe(CN)_6_] (10 g/L). The reaction was then left at 50 °C for 30 min. Afterwards, 0.25 mL trichloroacetic acid (100 g/L) was added to the reaction and centrifuged at 4,000 rpm for 10 min. Then, 0.075 mL of the supernatant was diluted with 0.075 mL of distilled water and 0.015 mL FeCl_3_ (1 g/L). The reaction was mixed and read at 700 nm. IC_50_ value was calculated at which the absorbance was 0.5. Lower IC_50_ shows higher reducing power.

#### 4.3.3. ABTS Radical Scavenging Assay

ABTS scavenging activity of essential oil was evaluated by the method of Tuyen et al. [39]. The preparation of samples was in the same manner as the DPPH assay. The ABTS solution was produced by adding 15 mL aqueous solution of ABTS 7mM with 240 µL potassium persulfate 140 mL. The mixture was kept at room temperature in the dark for 16 h. Prior to assay, the ABTS^+^ solution was diluted with MeOH to get an absorbance of 0.70 ± 0.05 at 734 nm. The reaction contained 0.15 mL of methanolic ABTS^+^ solution, and 0.018 mL of each essential oil (50–500 µg/mL in MeOH) was read at 734 nm after incubation for 30 min in the dark at room temperature. BHT (10-100 µg/mL) was used as positive control. The antioxidant of essential oil was expressed as follows:

ABTS (%) = [(A_control_ − A_sample_)/A_control_] × 100

A_control_ is the absorbance of reaction without essential oils; A_sample_ is the absorbance of ABTS radical with sample (essential oil, standard). The ABTS scavenging activity was examined by IC_50_ as inhibitory concentration of the essential oils for 50% reduction of ABTS.

#### 4.3.4. Antioxidant Activity using the β-Carotene Bleaching Test

The antioxidant of the essential oil was carried out by the method of Minh et al. [21] with some adjustments. An amount of 2 mg of β-carotene/linoleic acid was dissolved in 10 mL of chloroform and then 1 mL of the chloroform solution was added to 20 μL of linoleic acid and 200 mg of Tween-40. The mixture was evaporated by using a vacuum at 45 °C, then 50 mL pure oxygenated water was added and shaken vigorously to form an emulsion. An amount of 0.012 mL of essential oil (1 mg/mL in MeOH) was mixed with 0.1 mL of the emulsion. The reactions were left at 50 °C and read at 492 nm using a microplate reader (MultiskanTM Microplate Spectrophotometer, Thermo Fisher Scientific, Osaka, Japan). All reactions were read at zero time and every 15 min up to 180 min. Lipid peroxidation inhibition (LPI) was expressed as the following formula:

LPI (%) = A_1_/A_0_ × 100

A_0_ is the absorbance value measured at zero time for the essential oil, A_1_ is the absorbance measured after incubation for 180 min. Higher LPI value indicates the higher antioxidant activity.

### 4.4. Growth Inhibitory Activity Bioassays

Growth suppressing potential of EO from rice leaf was assayed on *Oryza sativa* (var. Koshihikari), *Echinochloa crus-galli*, *Bidens pilosa* L., *Raphanus sativus* L., and *Lactuca sativa* L. seeds in a growth chamber (Biotron NC system, Nippon Medical & Chemical Instrument, Co. Ltd., Osaka, Japan). Photoperiodic was set up at day/night 12/12 h with a temperature 25/23 °C. The EO sample was dissolved in water containing 0.2% of Tween 20 to obtain different concentrations (10, 100, 500, 1000, and 2000 µg/mL in MeOH). The test solution (100 µL) was permeated filter papers lined in a 12 well-plate (each well has 22 mm diameter x 18 mm height). Each healthy seed was then placed in a well, followed by the addition of 100 µL of distilled water. Plant germination monitoring was performed every 24 h for seven days. This bioassay was replicated six times (*n* = 6). The growth parameters of radicle (root) and hypocotyl (shoot) length were measured. Concentration in reducing 50% shoot and root lengths (IC_50_) was also calculated using a method described previously [40].

### 4.5. Xanthine Oxidase Inhibition (XOI) Activity

The inhibitory effect on xanthine oxidase (XO) of rice leaf EO was measured spectrophotometrically according to the method reported previously [41] with minor modifications. The mixture assay consisted of a 50 µL sample solution (2000, 1000, 500, 250, 125, and 62.5 µg/mL in DMSO), 30 µL phosphate buffer (70 mM, pH = 7.5), and 30 µL fresh enzyme solution (0.1 units/mL in the same buffer). The mixture assay then pre-incubated at 25 °C for 15 min before the main reaction conducted. The main reaction was initiated by adding 60 µL of substrate solution (150 µM xanthine in the same buffer) and then was incubated at 25 °C for 30 min. The reaction was stopped by adding 25 µL HCl (1 M), and the absorbance was recorded at 290 nm using a microplate reader. In this assay, allopurinol (6.25, 12.5, 25, 50 µg/mL) was used as a reference. A blank was prepared in the same manner, but the enzyme solution was added after HCl. XO inhibitory activity was expressed as the percentage inhibition of XO and calculated as following the formula:

$$\%\;\mathrm{Inhibition}\;=\;\{\frac{\left(A-B\right)-\left(C-D\right)}{\left(A-B\right)}\}\;\times \;100$$

where A is the absorbance of the enzyme without tested samples, B is the control of A without enzyme. C and D are the absorbances of the test solutions with and without XO, respectively. The IC_50_ values were calculated from the mean values of the data.

### 4.6. Identification of Volatile Compounds from Rice Leaf Essential Oil 

The compounds from EO of rice leaf were analyzed by GC-MS system (JMS-T100 GCV, JEOL Ltd., Tokyo, Japan) equipped with a DB-5MS capillary column, 30 m in length, 0.25 mm internal diameter, and 0.25 µm in thickness (Agilent Technologies, J & W Scientific Products, Folsom, CA, USA). The column temperature was setup initially at 50 °C without hold time, followed by an increase to 300 °C with the gradients of 10 °C/min and hold for 20 min. The temperature of the injector and detector were programmed respectively at 300 °C and 320 °C, with a mass scan range of 29–800 amu. The compounds were determined by the comparison between their mass spectral fragmentation pattern with the mass spectral libraries of JEOL’s GC-MS Mass Center System Version 2.65a. The compounds with high purity were selected for further spectroscopic techniques to structure elucidation [42].

ESI-MS analysis was conducted on a negative/positive ion mode. Mass spectral characterization was performed using a LTQ Orbitrap XL mass spectrometer (Thermo Fisher Scientific, San Jose, CA, USA) connected with an electrospray ionization (ESI) source in the negative (between m/z 120 and 2000) and positive (between m/z 100 and 2000) ionization mode recording spectra. The instrumental conditions were as follows: Spray voltage, 5.0 kV; sheath gas flow, 50 arb (arbitrary unit); aux gas flow rate, 10 arb; capillary temperature, 330 °C; capillary voltage, 50 V; tube lens, 80 V [43].

APCI-MS analysis was implemented using a mass spectrometer with an electrospray ion source. Mass spectral characterization was also performed using an LOQ Orbitrap XL mass spectrometer. The measurements were conducted on the positive mode (*m/z* 100–2000) FTMS at a resolution of 60,000. The instrumental conditions were as follows: Source voltage, 3.5 kV; source current, 6.12 uA; vaporizer temperature 401 °C; sheath gas flow, 20 arb; aux gas flow rate, 10 arb; capillary temperature, 250 °C; capillary voltage, 30 V; tube lens, 80V [44].

### 4.7. Identification and Quantification of Momilactones A and B from EO of Rice Leaf by UPLC/ESI-MS

The characterization of major component peaks of EO from rice leaf was performed on the Waters Acquity UPLC instrument equipped with the Acquity HPLC BEH C18 1.7 µm (2.1x50 mm Column) (Waters Cooperation, Milford, MA, USA). The UPLC mobile phases were (A) 0.1% formic acid in water (*v*/*v*) and (B) 0.1% formic acid in acetonitrile (*v*/*v*). Isocratic elution was accomplished with a mixture of A 50% and B 50%. The flow rate was 0.3 mL/min, injection volume was 3.0 μL, and the column temperature was 30 °C, with an ambient sample temperature. Mass spectral characterization was performed using an LOQ Orbitrap XL equipped with an electrospray ionization source in positive ionization mode recording spectra between m/z 100 and 1000. The instrumental conditions were as follows: Spray voltage, 4.5 kV; sheath gas flow, 55 arb; aux gas flow rate, 15 arb; capillary temperature, 340 °C; capillary voltage, 50 V; tube lens, 80V [45].

## 5. Conclusions

Findings of this study not only revealed the chemical profile but also revealed the potential antioxidant, anti-hyperuricemia, and plant growth inhibitory activities of EO from rice leaves. There were twelve volatile constituents identified, of which methyl ricinoleate (27.86%) and palmitic acid (17.34%) were the principal compounds. This study was the first to successfully identify the presence of momilactones A and B by ultra-performance liquid chromatography coupled with electrospray mass spectrometry (UPLC/ESI-MS), suggesting that they may be involved in plant growth inhibition as well as other biological activities of rice EO. Because rice EO exhibited promising inhibition on the two noxious weeds of *E. crus-galli* anb *B. pilosa*, further trials on examining individual volatile compounds as well as momilactones A and B on the two weed species should be further elaborated. It was observed that the EO of rice leaf was beneficial as a source of antioxidants and a reduction of gout disease, and thus provided extra benefits for rice farmers in developing countries. However, the use of lower temperatures during evaporation should be further conducted to enhance the actual yield of EO in rice leaves.

## Figures and Tables

**Table 1:** Antioxidant activity measured by DPPH, ABTS, FRAP, β-carotene bleaching assays, and XOI activity of rice leaf EO in terms of IC_50_ values.

Sample	IC_50_ (µg/mL)			LPI (%)	IC_50_ (µg/mL) XOI
	DPPH	ABTS	FRAP	β-carotene	
Rice leaf EO	73.1 ± 1.4	198.3 ± 2.2	700.8 ± 5.7	79.0%	526.0 ± 2.3
BHT *	9.3 ± 1.1	45.7 ± 1.4	426.7 ± 3.8	90.0%	-
Allopurinol *	-	-	-	-	21.5 ± 0.2

* Positive control. -: measurements were not conducted. Data are means ± SD (standard deviation) (*n* = 3).

**Table 2:** Inhibitory effects of rice leaf EO on the growth of *O. sativa*, *E. crus-galli*, *B. pilosa*, *R. sativus*, and *L. sativa*

Plant Species	Inhibition Percentage (%)						IC_50_ (µg/mL)	
	Root			Shoot				
	100 *	500 *	1000 *	100 *	500 *	1000 *	Root	Shoot
*Oryza sativa* L.	−10.0	−12.0	−3.0	−5.0	−7.0	−11.0	St **	St **
*Echinochloa crus-galli*	38.0	51.0	63.0	18.0	25.0	51.0	455.6 ± 11.5 b	964.3 ± 12.1 a
*Bidens pilosa* L.	12.0	20.0	52.0	16.0	19.0	56.0	912.5 ± 10.3 a	869.2 ± 6.1 bc
*Raphanus sativus* L.	4.0	21.0	60.0	7.0	20.0	60.0	916.3 ± 10.0 a	866.2 ± 26.1 c
*Lactuca sativa* L.	6.0	34.0	55.0	12.0	24.0	55.0	926.7 ± 11.6 a	908.0 ± 11.1 b

* Concentration of EO (µg/mL). ** Stimulation. Data are presented as means ± SD (standard deviation). Mean values with different lowercase letters indicate significant differences in the same column (*p* < 0.05) (*n* = 3).

**Table 3:** Identification of phytochemicals in essential oil of rice leaf by GC-MS, confirmation by ESI-MS and APCI-MS.

Chemical Formula	Compounds	Molecular Weight	Retention Time (min)	Peak Area [% of Total]
C_8_H_8_O	Coumaran	120	8.22	4.02
C_9_H_10_O_2_	Benzyl acetate	150	9.58	2.54
C_8_H_8_O_3_	Vanillin	152	10.73	8.22
C_11_H_22_O_2_	Undecanoic acid	186	12.73	2.56
C_13_H_18_O	Megastigmatrienone	190	13.02	3.20
C_14_H_28_O_2_	Myristic acid	228	15.00	3.26
C_18_H_36_O	2-Pentadecanone	268	15.89	2.13
C_10_H_20_O_2_	Capric acid	172	16.05	2.17
C_16_H_32_O_2_	Palmitic acid	256	17.13	17.34
C_18_H_30_O_2_	Linolenic acid	278	18.80	11.16
C_19_H_36_O_3_	Methyl ricinoleate	312	19.46	27.86
C_16_H_34_	Hexadecane	226	20.22	3.82

**Table 4:** Identification and quantification of momilactones A and B from rice leaf EO by UPLC/ESI-MS.

Rice leaf EO	UPLC/ESI-MS	
	MA	MB
Retention time (min)	4.00 ± 0.04	2.45 ± 0.06
LOD (ng/mL)	0.097	0.157
LOQ (ng/mL)	0.293	0.476
Yield (ng/g FW)	9.80 ± 0.22	4.93 ± 0.13
% of Total EO	7.35	3.70

FW: Fresh weigh. Data are means ± SD (standard deviation) (*n* = 3). MA: Momilactone A; MB: Momilactone B.

## Supplementary Materials

The following are available online, Figures S1–S5a,b were provided.

## References

[1] Farmer E.E. (2001). Surface-to-air signals. Nature.

[2] Kask K., Kännaste A., Niinemets Ü. (2013). Emission of volatile organic compounds as a signal of plant stress. Sci. Bull. ESCORENA.

[3] Villamar-Torres R., Mehdi S., Liuba-Delfini G., García L., Viot C.R. (2018). Volatile organic compounds: Plant natural defense mechanisms against herbivorous arthropods and an opportunity for plant breeding of cotton. Sci. Agric..

[4] Djilani A., Dicko A., Bouayed J. (2012). The therapeutic benefits of essential oils. Nutrition, well-being and Health.

[5] Pickett J.A., Khan Z.R. (2016). Plant volatile-mediated signaling and its application in agriculture: Successes and challenges. New Phytol..

[6] Cha H.M., Han G., Chung H.J. (2012). A study on the trend analysis regarding the rice consumption of Korean adults using Korean National Health and Nutrition Examination Survey data from 1998, 2001 and 2005. Nutr. Res. Pract..

[7] Champagne E.T. (2008). Rice aroma and flavor: A literature review. Cereal Chem..

[8] Wang W., Li Y., Dang P., Zhao S., Lai D., Zhou L. (2018). Rice secondary metabolites: Structures, roles, biosynthesis, and metabolic regulation. Molecules.

[9] Suzuki Y., Ise K., Li C., Honda I., Iwai Y., Matsukura U. (1999). Volatile components in stored rice [*Oryza sativa* (L.)] of varieties with and without lipoxygenase-3 in seeds. J. Agric. Food Chem..

[10] Lin J.Y., Fan W., Gao Y.N., Wu S.F., Wang S.X. (2010). Study on volatile compounds in rice by HS-SPME and GC-MS. Julius-Kühn-Archiv..

[11] Yang D.S., Lee K.S., Jeong O.Y., Kim K.J., Kays S.J. (2007). Characterization of volatile aroma compounds in cooked black rice. J. Agric. Food Chem..

[12] Yang D.S., Shewfelt R.L., Lee K.S., Kays S.J. (2008). Comparison of odor-active compounds from six distinctly different rice flavor types. J. Agric. Food Chem..

[13] Sukhonthara S., Theerakulkait C., Miyazawa M. (2009). Characterization of volatile aroma compounds from red and black rice bran. J. Oleo Sci..

[14] Liyanaarachchi G.D., Kottearachchi N.S., Samarasekera R. (2014). Volatile profiles of traditional aromatic rice varieties in Sri Lanka. J. Natl. Sci. Found. Sri Lanka.

[15] Yajima I., Yanai T., Nakamura M., Sakakibara H., Habu T. (1978). Volatile flavor components of cooked rice. Agric. Biol. Chem..

[16] Kongkiattikajorn J. (2008). Effect of storage time and temperature on volatile aroma compounds and physicochemical properties of rice. Kasetsart J. Nat. Sci..

[17] Kim H.R., Kim K.M., Woo K., Jeong H.S., Kim K.O. (2015). Changes in volatile compounds of waxy rice and *gangjeong* (a traditional Korean oil-puffed snack) under different steeping conditions. Food Sci. Biotechnol..

[18] Sirisantimethakom L., Laopaiboon L., Danvirutai P., Laopaiboon P. (2008). Volatile compounds of a traditional Thai rice wine. Biotechnology.

[19] Lee S.M., Han H.Y., Lee S.J. (2014). Volatile compounds in takju (rice wine) using different types of fermentation starters. Food Eng. Prog..

[20] Minh T.N., Xuan T.D., Ahmad A., Elzaawely A.A., Teschke R., Van T.M. (2018). Momilactones A and B: Optimization of yields from isolation and purification. Separations.

[21] Minh T.N., Xuan T.D., Ahmad A., Elzaawely A.A., Teschke R., Van T.M. (2018). Efficacy from different extractions for chemical profile and biological activities of rice husk. Sustainability.

[22] Laosinwattana C., Wichittrakarn P., Teerarak M. (2018). Chemical composition and herbicidal action of essential oil from *Tagetes erecta* L. leaves. Ind. Crops Prod..

[23] Tsasi G., Mailis T., Daskalaki A., Sakadani E., Razis P., Samaras Y., Skaltsa H. (2017). The effect of harvesting on the composition of essential oils from five varieties of *Ocimum basilicum* L. cultivated in the island of Kefalonia, Greece. Plants.

[24] Ahmad A., Xuan T.D., Minh T.N., Siddiqui N.A., Quan N.V. (2019). Comparative extraction and simple isolation improvement techniques of active constituents’ momilactone A and B from rice husks of *Oryza sativa* by HPLC analysis and column chromatography. Saudi Pharm. J..

[25] Sefidkon F., Abbasi K., Khaniki G.B. (2006). Influence of drying and extraction methods on yield and chemical composition of the essential oil of *Satureja hortensis*. Food Chem..

[26] Božović M., Navarra A., Garzoli S., Pepi F., Ragno R. (2017). Esential oils extraction: A 24-h steam distillation systematic methodology. Nat. Prod. Res..

[27] Giacometti J., Kovačević D.B., Putnik P., Gabrić D., Bilušić T., Krešić G., Stulić V., Barba F.J., Chemat F., Barbosa-Cánovas G. (2018). Extraction of bioactive compounds and essential oils from mediterranean herbs by conventional and green innovative techniques: A review. Food Res. Int..

[28] Amic D., Davidovic-Amic D., Beslo D., Rastija V., Lucic B., Trinajstic N. (2007). SAR and QSAR of the antioxidant activity of flavonoids. Curr. Med. Chem..

[29] Toscano-Garibay J.D., Arriaga-Alba M., Sánchez-Navarrete J., Mendoza-García M., Flores-Estrada J.J., Moreno-Eutimio M.A., Espinosa-Aguirre J.J., González-Ávila M., Ruiz-Pérez N.J. (2017). Antimutagenic and antioxidant activity of the essential oils of *Citrus sinensis* and *Citrus latifolia*. Sci. Rep..

[30] Bassolé I.H.N., Juliani H.R. (2012). Essential oils in combination and their antimicrobial properties. Molecules.

[31] Miyazawa M., Nagai S., Oshima T. (2008). Volatile components of the straw of *Oryza sativa* L.. J. Oleo Sci..

[32] Oloyede G.K. (2012). Antioxidant activities of methyl ricinoleate and ricinoleic acid dominated *Ricinus communis* seeds extract using lipid peroxidation and free radical scavenging methods. Res. J. Med. Plant.

[33] Karimi E., Jaafar H.Z., Ghasemzadeh A., Ebrahimi M. (2015). Fatty acid composition, antioxidant and antibacterial properties of the microwave aqueous extract of three varieties of *Labisia pumila* Benth. Biol. Res..

[34] Kato-Noguchi H. (2011). Convergent or parallel molecular evolution of momilactone A and B: Potent allelochemicals, momilactones have been found only in rice and the moss *Hypnum plumaeforme*. J. Plant Physiol..

[35] Xuan T.D., Minh T.N., Anh L.H., Khanh T.D. (2016). Allelopathic momilactones A and B are implied in rice drought and salinity tolerance, not weed resistance. Agron. Sustain. Dev..

[36] Charles D.J., Simon J.E. (1990). Comparison of extraction methods for the rapid determination of essential oil content and composition of basil. J. Am. Soc. Hortic. Sci..

[37] Minh T.N., Khang D.T., Tuyen P.T., Minh L.T., Anh L.H., Quan N.V., Ha P.T.T., Quan N.T., Toan N.P., Elzaawely A.A. (2016). Phenolic compounds and antioxidant activity of *Phalaenopsis* orchid hybrids. Antioxidants.

[38] Minh T.N., Tuyen P.T., Khang D.T., Quan N.V., Ha P.T.T., Quan N.T., Yusuf A., Fan X., Van T.M., Khanh T.D. (2017). Potential use of plant wastes of moth orchid (*Phalaenopsis* Sogo Yukidian ‘V3′) as an antioxidant source. Foods.

[39] Tuyen P.T., Xuan T.D., Khang D.T., Ahmad A., Quan N.V., Anh T.T.T., Anh L.H., Minh T.N. (2017). Phenolic compositions and antioxidant properties in bark, flower, inner skin, kernel and leaf extracts of *Castanea crenata* Sieb. et Zucc. Antioxidants.

[40] Xuan T.D., Tsuzuki E., Terao H., Matstuo M., Khanh T.D., Murayama S., Hong N.H. (2003). Alfalfa, rice by-products and their incorporation for weed control in rice. Weed Biol. Manag..

[41] Nguyen M.T.T., Awale S., Tezuka Y., Tran Q., Le Watanabe H., Kadota S. (2004). Xanthine oxidase inhibitory activity of Vietnamese medicinal plants. Biol. Pharm. Bull..

[42] Van T.M., Xuan T.D., Minh T.N., Quan N.V. (2018). Isolation and purification of potent growth inhibitors from *Piper methysticum* root. Molecules.

[43] Banerjee S., Mazumdar S. (2012). Electrospray ionization mass spectrometry: A technique to access the information beyond the molecular weight of the analyte. Int. J. Anal. Chem..

[44] Kim Y.H., Kim S. (2010). Improved abundance sensitivity of molecular ions in positive-ion APCI MS analysis of petroleum in toluene. J. Am. Soc. Mass Spectr..

[45] Prokudina E.A., Havlíček L., Al-Maharik N., Lapčík O., Strnad M., Gruz J. (2012). Rapid UPLC-ESI-MS/MS method for the analysis of isoflavonoids and other phenylpropanoids. J. Food Compos. Anal..

